# Chronomobility of international students under COVID-19 Australia

**DOI:** 10.3389/fsoc.2023.1159600

**Published:** 2023-12-08

**Authors:** Surjeet D. Dhanji, Jun Ohashi, Jay Song

**Affiliations:** Asia Institute, Faculty of Arts, The University of Melbourne, Parkville, VIC, Australia

**Keywords:** international students, chronomobility, COVID-19, Australia, coping mechanism

## Abstract

This article investigates the chronomobility of international students in Australia going through COVID-19. Existing literature on international students approaches them largely in two manners: a market or victims. Using Shanti Robertson’s chronomobility, the study focuses on international students’ coping mechanisms and strategies for their next moves. Drawing from 15 in-depth interviews with international students formally enrolled in Australian institutions in Melbourne, the longest lockdown city during the pandemic, the authors find various ways of short-term coping mechanisms through meditation, physical exercises, virtual escapism and counselling. Furthermore, despite pandemic immobility, students presented a high level of resilience in making future decisions for post-pandemic mobilities. We conclude that family support and social networks are key to realise full potentials of international students as skilled migrants and valued members of society. Our manuscript contributes to the field of migration and mobility by enriching Robertson’s concept of chrono-mobility and adding the empirical case study from international students in Australia during the latest pandemic in 2020–2021.

## 1 Introduction

COVID-19 exposed the weakest links in society and impacted the most vulnerable populations harder than it did others. As temporary migrants, international students are one of these vulnerable groups. This study aims to investigate the chronomobility of international students in Australia.

The concept ‘chronomobilities’ describes the temporalities that structure mobile lives, but also how these mobile lives emerge from these temporalities, via a conceptual framework of ‘time-regimes’—the macro and mesoscale temporal conditions that shape contemporary social life—and ‘time-logics’—the way individuals narrate and make meaning of their lived experiences of time (Robertson, 2021).

Robertson’s chronomobility framework works through the complexities of the relationship between migrant temporalities and migrant mobilities that guide researchers in understanding two issues: one relationship; and two, the experiences it produces in migrants’ lives. This paper captures Robertson’s Chronomobility’ concept of temporariness and expands it by demonstrating how migrant temporalities and temporary status extends even as national geographical locations are shifting. Much like a pendulum, these transnational students are moving from location to location to reach Australia within the tight stipulated government time deadlines, but their status remains in a flux. Although chronomobility provides a better tool to adequately grasp experiences of international students during a period in which their migration is both unsettled and continuous in a reality that is ‘middling migration’, the concept has limitations for this paper that is based on 3 years from 2020 to 2022. This paper forms the foundation basis on the study of international students as the post-COVID-19 or ‘aftershocks’ on international students and international higher education are on-going research.

In particular, it intends to answer the following questions: (1) how the pandemic affected international students as potential future skilled migrants in Australia? and (2) how international students illuminated both their challenges and coping mechanisms in the context of Melbourne, including a wide range of pandemic-related racism, economic difficulties, and psychological anxiety, but also micro-strategies for their future life plans.

**Figure 1:**
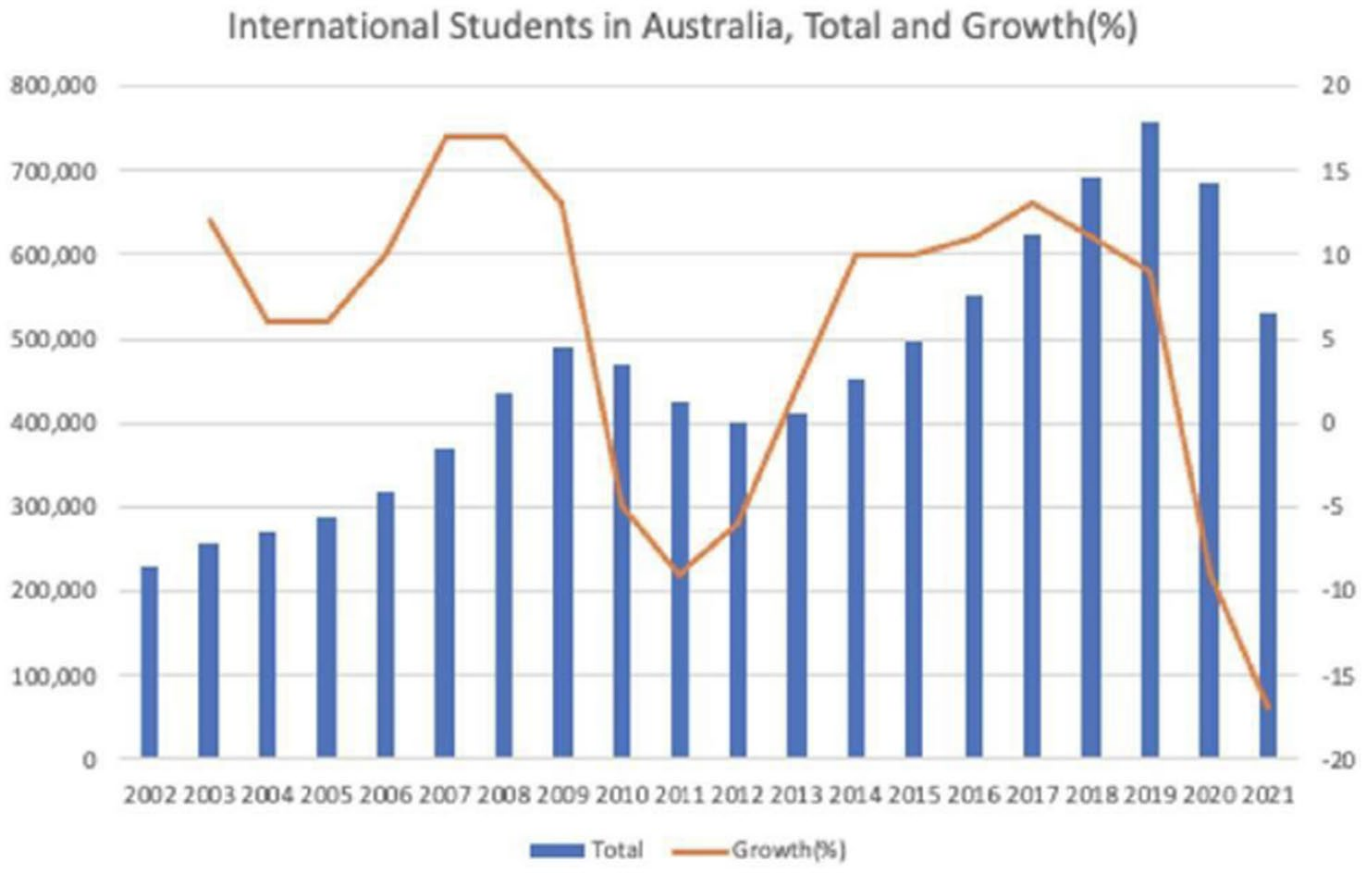
International students in Australia, total and growth (%). Source: Australian Department of Education, Skills and Employment at https://internationeducation.gov.au/research/DataVisualisations/Pages/Student-number.aspx, accessed 6 February 2023.

**Figure 2:**
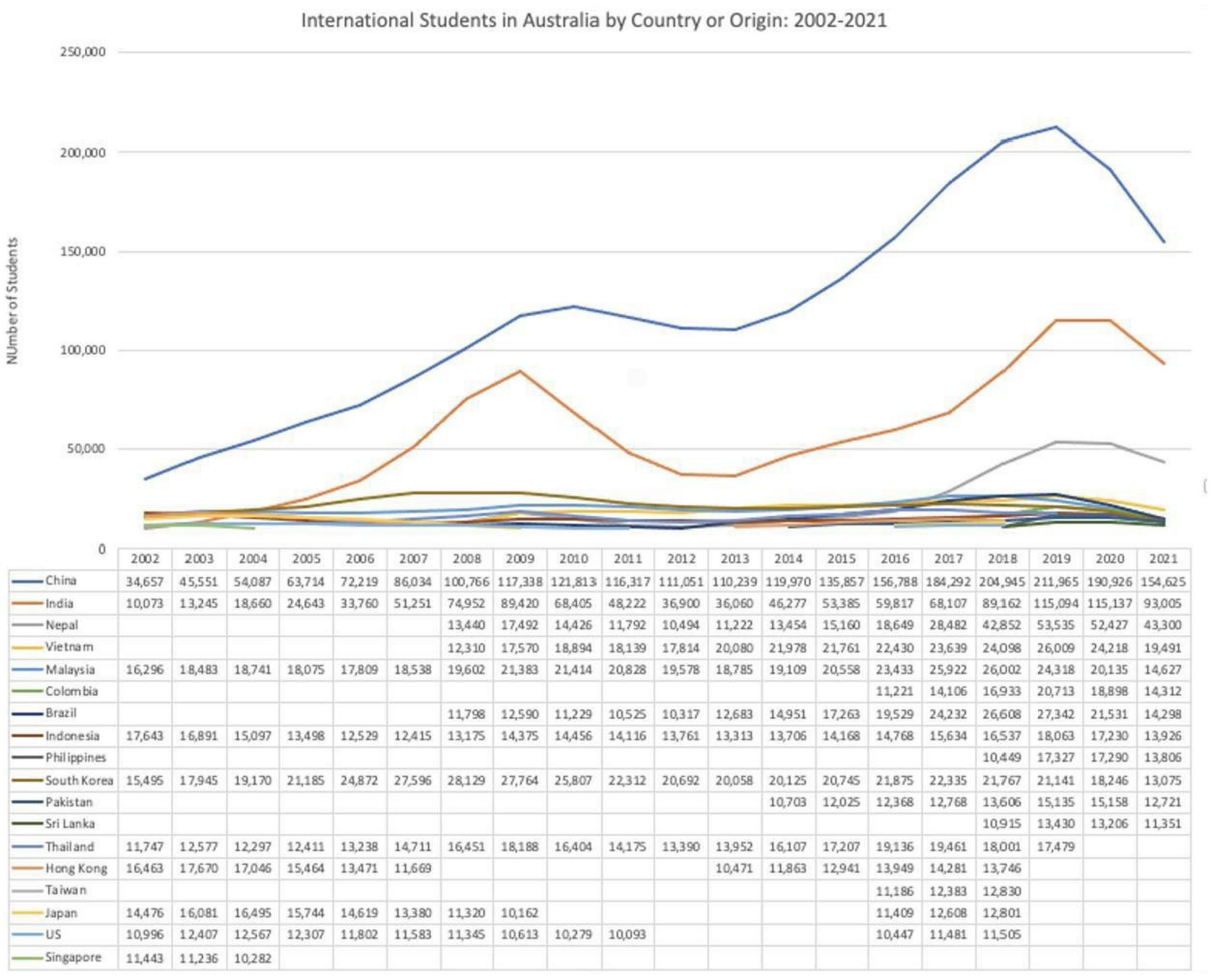
International students in Australia by country of origin (2002–2021). Source: Australian Department of Education, Skills and Employment at https://internationeducation.gov.au/research/DataVisualisations/Pages/Student-number.aspx, accessed 6 February 2023.

Figure 1 shows the numbers of international students in Australia over the past two decades. Figure 2 indicates that China and India have been the top two source countries since 2005. After the global financial crisis in 2009, the numbers from both China and India dropped significantly. However, from 2013 onward numbers increased steadily until 2016. When the pandemic hit in 2020, the decline struck harder, with the total growth rates in 2020 and 2021 displaying negative growth of −9% and −17%, respectively, amongst students from China and India. Other than the top two source countries, Malaysia, Indonesia, and South Korea have been major source countries of students for the Australian higher education sector. In the past two decades, Taiwan, Hong Kong, Japan, and the US ceased to be major source countries, but Nepal has been the fastest growing source country in recent years. South Asia and Latin America, too, have become important providers: numbers from Pakistan, Sri Lanka, Colombia, and Brazil have all increased over that time. What is significant about these shifting numbers and source countries is that they show future trends in skilled migration, permanent residency and Australia’s demographic changes.

In the following section, we first critically review the current literature on international student migration, followed by a description of the data and methodology. Next, we elaborate the findings on emerging themes of pandemic racism, economic vulnerability, and coping mechanisms and strategies. We argue that existing approaches international students should be expanded to see them as a future skilled workforce, rather than as a profitable market or victims of temporary labour migrants from a migration and population perspective. Today’s international students can be valued members of future Australian society.

## 2 International student migration: a critical review

The existing literature on international students offers two polarised views on the subject. A prevalent economic perspective views them as ‘cash cows’ for domestic educational sectors. International students are objectified as lucrative markets. Another view treats them as victims of marginalisation and potential exploitation. International students are often perceived as subjects with reduced agency and resilience. Few studies have discussed the adaptation, learning and sense of belonging experienced by international students during the pandemic in Australia and expressed in their own voices.

### 2.1 International students as a market

Assessing the supply and demand in international student migration, Findlay (2011) examines changing characteristics of international student mobility. Findlay’s social demand theory explains the power of socio-cultural capitals that drive middle-class Asian families to send their children to English-speaking universities in the West. However, Open Doors (2005) and HESS (2008) supply-side theorists and researchers argue that, to a substantial extent, the global flows of students are powered by the financial interests of those who can deliver elite higher education opportunities to the market in Asia. In similar vein, Abbott and Silles’ (2016) gravity model finds that geographical distance, time zone difference and the presence of a common language are powerful economic explanations for student mobility as they gravitate towards education destinations. The gravity models provide insights into the current trends influencing and motivating enrolments and explanations for the rapidly increasing numbers of Asian international student mobility to Australia. The relevance of student mobility – the push and pull demand and supply and gravity models—is a prime mechanism for marketing international education by underscoring how studying abroad by students can enhance their ability to gain skills and an international qualification that yield economic benefits to the students.

Yet drawing these together, and using OECD statistics on international student migration, King and Raghuram’s (2013) *International Student Migration: Mapping the Field and New Research Agendas* study notes that research on international students is understudied amongst migration scholars. The study highlights the material vs. societal contradiction in dealing with international student populations. On the financial side, the internationalisation of education leads to dependence on substantial tuition fees from international students being ‘desired’, which contribute to building revenues for domestic education sectors. On the social and societal side, the gravity model views them only as temporary migrants, or ‘cash cows’ and outsiders, who are ‘unwanted’ in the longer term as residents.

Still, the profitability of higher education remains a primary concern. For instance, Ahlburg (2020) and Burki (2020) analyse the significant losses to higher education in the UK because of COVID-19. In February 2021, Universities Australia were concerned that universities had lost AU$1.8 billion in revenue for 2020 and would be facing an AU$2 billion loss in 2021. Similarly, Firang’s (2020) study exploring the effects of the pandemic on Universities in Canada, finds the decline in international students’ admission was creating economic implications for Canadian universities. Overall, there is scant literature on how global and national crises, such as the pandemic, impact international students, are limited.

Similarly, Gamlen (2020) links the future profitability of ‘export education’ as a factor in deciding international student migration: Gamlen’s report to the International Organisation for Migration on the impact of COVID-19 on migration and mobility predicts that migration will fall further before bouncing back. Similarly, Jayasuriya’s (2021) regulatory model of market citizenship as a causal factor in worsening the effect of COVID-19 on Australia’s higher education sector pinpoints strong marketisation towards and dependence on international students.

Ross (2020) offers insights on the economic ramifications of COVID-19 for higher education, arguing that Australian universities are financially dependent on income from international students, which has been undermined by COVID-19. Exploring how Australia mitigates the loss of profits owing to COVID-19’s effect on international student migration into Australia, Haugen and Lehmann (2020) specifically scrutinise how Chinese students (65% of the international student cohort) were travelling through third countries as ‘clearing houses’ to enter Australia. They highlight the Australian federal government’s strategic involvement in facilitating this emerging trend which served as a means to generate essential and prevent potential losses.

### 2.2 International students as victims

A contrasting approach to international students comes from human rights and social work experts. This perspective identifies problems with government policies on international students, analysing the impact on students and on education sectors. Common factors include inadequate government responses to COVID-19, the continuing effects of past restrictive immigration policies on international students, and the emerging social discrimination against international students. For example, Nguyen and Balakrishnan (2020) criticise the Australian government’s decision in March 2020 to refuse to provide international students with adequate support for mental health, financial difficulties, and exploitation. In Canada, too, Firang (2020) and Macklin (2020) report that social workers examining on impact of the pandemic found that an inadequate Emergency Response Benefit for international students at the beginning of the pandemic could have long-term effects on Canada’s education sectors (Firang, 2020; Macklin, 2020). Overall, these studies point to the long-term effects on international students caused by financial hardships and racism under the pandemic in 2020 (Welch, 2020; Fronek et al., 2021). In the early phase of the pandemic in Australia, racist phrases such as ‘China virus’ and the ‘Kung-flu’ shouted towards Asians were becoming intensified coupled by the deteriorating geo-political relations and trade issues between China and Australia (Razik, 2020; Zhou, 2020; Tan et al., 2021). Racism—sometimes implicit and specifically conveyed by anti-Asian sentiments—and at other times nuanced such as crossing the road over when encountering Asians—itself during the pandemic.

### 2.3 International students mobility constraints

There is also a growing body of research that seeks to understand the lived experiences of international students during the pandemic from their own perspective. For example, Mok et al.’s (2021) recent survey on international students examined the impact of COVID-19 on student mobility in China and Hong Kong. The authors found that the pandemic has not only decreased international student mobility, but it also shifted the flow of international students, changing their global migratory routes and destinations. Of the 2,739 respondents studied, 84% showed no interest to study abroad after the pandemic. For those who still wished to pursue overseas degrees, apart from the US and the UK, Hong Kong, Japan and Taiwan were listed as the top five destinations. Australia used to be listed high but was being ranked down during COVID-19 amongst Chinese students (Xiong et al., 2020; Wang and Crawford, 2021). The shift is in part weighing the management of the COVID-19 crisis by Australia with international students’ desiring to remain in close proximity to Mainland China—in Hong Kong, Japan and Taiwan—with better health measures for exposure and availing easy retreat to homeland when necessary (Mok et al., 2021).

The authors conclude that the global health crisis will intensify socio-economic inequalities and regionalism in international student migration mobility given that China is a valuable source country for international students to the region.

### 2.4 International students’ active agency during (im)mobility

Focussing on the relationship between international student mobility and transnational parent–child relations, Hu et al. (2022) examined how Chinese international students in the UK and their parents in China navigated transnational mobilities during the pandemic. Based on interviews with 16 students and 8 parents, the study found that both students and their parents made active decisions on whether to remain in the UK or return to China focussing on ‘other’ concerns. Factors such as aligning available resources and cost–benefit analyses, as well as the ‘emotional work’ involved in these processes. This study also found a significant shift from dependence on government institutional infrastructures to family-mediated private infrastructures for transnational migration. ‘Other’ concerns refer to parents modifying their emotive expressions by downplaying worries and concerns to maintain a sense of normalcy.

Shifting from demand and supply, and gravity model theories that are viewing international students through the ‘education commodity’ lens as a major source of revenue for universities and tertiary education (as objects), on the one hand, and viewing them as victims of exploitation, racism and human rights violations, on the other, and as subjects, does not appreciate their agency and complex experiences of time in (im)mobilities during the pandemic. The rising tide of migration governance and transification of migrants in the 21st century—particularly during the pandemic—has shown up some of the newer challenges that are yet to be theorised.

Existing framework in the broad field of migration studies, cuts across single disciplines as well as interdisciplinary disciplines and spaces of ethnic and racial studies. Yet the past decade—including the ‘migration governance in Australia as one of the transification of migration’ (p: 46) including the pandemic period—has seen the emergence of a ‘temporal turn’ that leads scholars to rethink migration experiences in the 21st century. Chronomobility provides a tool that extends beyond existing understandings of demand–supply, gravity models to adequately grasp experiences of international students during a period in which their migration is both unsettled and continuous in a reality that is ‘middling migration’ (Robertson, 2021). As middling and often precarious transnational subjects, international students imagine how their accumulated cultural and social capital lends to envisioning a better future despite their precarious status during the pandemic. On the one hand these transnational students they are trapped during the COVID-19 with international border and campus closures affecting their mental health negatively. On the other hand, they are resourceful and come up with solutions, epistemological imagining to cope with their situation.

Robertson’s (2021, p. 26) argument that a new framework is necessary to understand mobility of Asian international students, who have ‘more social and economic resources’ than ‘low skilled workers and undocumented or forced migrants’. Whilst this ‘middling status’ may inspire aspiration for upward mobility and a positive lifestyle, their lived life entails a series of contingencies: from unexpected hardships, short-time sacrifices, to changes in their outlook on life within a temporal–spatial migration framework. Robertson’s ‘middling’ class is a different concept from middle class as it stresses the ongoing and transitional process of life courses of young migrants. This has given rise to asking new interrelated questions on migrant temporalities and mobilities and the affective experiences in the day to day lives of international student migrants who are ‘temporary migrants’ and as such fall into the migrant temporality—outsiders or amateurs—and migrant mobility categories.

Other authors present similar arguments. Moving from the focus on perspectives and resources of international students, Berg et al.’s (2020) Report, As If We Weren’t Humans: The Abandonment of Temporary Migrants in Australia During COVID-19, outlines the Australian government’s failure to provide essential support to temporary migrants, neglecting its moral obligations to community members—transitioning between different temporary visas—whom it encouraged to greatly invest in studying and working in Australia. This Report emphasises the temporality of migrants. Jokila et al. (2022) in Reimagining Globalisation and Education argue that in the past decade, student mobility has surged unforeseen numbers globally under new policy and funding initiatives, but that ongoing crisis such as the pandemic and climate change, challenge rethinking afresh the recurring mobility motives. Robertson gathers these new challenges, compelling scholars to reimagine the relationship or links between migrant temporality and migrant mobility through the concept of chronomobility.

Robertson’s perspective, termed ‘chronomobility’, offer a better framework for understanding Asian international students’ diverse reactions to the pandemic. This study aims to fill the knowledge gaps by, first, investigating international students’ reactions to the pandemic through a ‘chronomobility’ lens that allows us to capture the complex inter-relationship between time, space and agency during the pandemic, and second, by showing their diverse lived experiences and coping strategies to alleviate difficulties.

## 3 Materials and methods

An inter-disciplinary team of researchers from migration, human rights and sociolinguistics conducted in-depth interviews with 15 international students who were officially enrolled in Australian institutions—largely in metropolitan Melbourne, Victoria but also in metropolitan Canberra, Australian Capital Territory—as international students as at October 2021. Ethics approval was granted by Melbourne University prior to the interviews. We recruited students through existing networks and via the institution’s newsletter. The selection criteria were that students were to have diverse backgrounds of nationality, ethnicity, years of residence in Australia, the degree programme undertaken, and financial condition. The last category shows whether they are on scholarship or are funded by their parents. Each semi-structured interview took place via Zoom for 60–90 min. Pseudonyms were used to protect the privacy and confidentiality of the participants. Guiding questions largely included their day-to-day conditions, particular challenges they faced during the pandemic and copying mechanisms.

The demographic characteristics such as visa status criteria followed by year of arrival, age and country of origin, and current study-job status data were first analysed manually using Excel to identify interviewees’ ‘temporary’ status. The 15 interviewees (Table 1) were from Mainland China, South Korea, Vietnam, Indonesia, Malaysia, Hong Kong, Singapore, Philippines, Burma, India, Pakistan, Bangladesh and Mexico. They were enrolled in BA to PhD programmes in Australian institutions. Years of residence varied from two to 12 years to ascertain their ‘temporality’ and ‘mobility’ status. Like most Australian cities housing international students, all resided in Melbourne metropolitan areas, excepting two who were stranded overseas owing to border closures. Five were scholarship holders and the rest self-funded. Altogether the 15 interviewees represent a balanced target sample of the international student cohort at the time. All interviews were recorded with prior consent and then transcribed. Thereafter, coding analyses was done via NVivo on frequent words and word associations that reflected on relationships, between migrant temporalities and migrant mobilities links, between emotional experiences of time, day to day, in migrants’ work, leisure and social experiences reflected together with their concerns and a wide range of coping mechanisms to contextualise their chronomobility, that is, the reality of their transient experiences during the pandemic, which are discussed next.

**Table 1:** Interview participants.

Nationality	Ethnicity	Age	Education	Years of residence in Australia	Scholarship/finance	Pseudonym
Bangladesh	Bangla	28	MA	2	Partial/parents	Abir
Bangladesh	Bangla	28	MA	2	Parents	Ibrahim
Hong Kong	Chinese	28	MA	2	Parents	Elvina
Canada/Hong Kong	Chinese	24	MA	12	Parents	Su
Mainland China	Chinese	22	MA	2	Parents	Elise
India	Indian	23	MA	2	Parents	Amar
India	Indian	29	PhD	Stranded	Scholarship	Priya
Indonesia	Indonesian	19	BA	2.5	Parents	Kevin
Korea, South	Korean	30	PhD	11–12	Scholarship	June
Malaysia	Burmese	22	BA	3	Parents	Pam
Mexico	Latina	30	MA	4	Scholarship	
Pakistan	Pakistani	48	PhD	7	Scholarship	Shamim
Philippines	Philippines	28	PhD	2.5 Stranded	Scholarship	Gilbert
Singapore	Chinese-Peranakan	20	BA	3	Parents	Karmin
Vietnam	Vietnamese	21	JD	4	Parents	Huu

### 3.1 Pandemic racism and vulnerabilities

Several emerging themes transpired that can be divided into two broad categories. The first relate to the precarity of international students’ day to day living during the COVID-19 pandemic and the second relate to their efforts in finding some normalcy to endure the pandemic. International students were having to deal with their perceived ‘outsider’ and ‘temporary’ status, essential to the education export commodity but also dispensable. Added to this was the COVID-19 related racism and anti-Asian sentiments, the downturn of the economy and the shock of financial precarity and transitioning to digital learning. With no relief in immediate sight, international students were finding coping mechanisms and coping strategies for learning to live with uncertainty. They sought solace in both known and some newer ways of living with the reality for some normalcy in their lives.

On 3 April 2020, former Australian Prime Minister Scott Morrison said that temporary migrants, including international students, who could not support themselves, needed to go back to their countries or origin (Gibson and Moran, 2020). As international borders were closed and universities and shops had shuttered their blinds, researchers were able to mainly reach out to international students in Melbourne via social networks. Many of the interviewees felt alienated by Morrison’s statement. Amar said, ‘when the pandemic broke out, I remember the PM …made it very like blatantly… spelt it out… he said international students can go back’. Su says this kind of public messaging somehow ‘had become ingrained’ in international students, a constant reminder that ‘this was not our country’, and therefore they ‘learned not to expect anything’ from the government. Those sharing accommodation with domestic students found it hard to discuss their emotions with their housemates, feeling the latter would not grasp how international students felt: ‘[Their experiences] were different from mine. [We were] outsiders even though we have been here for 10 years and have our own social networks’ (Priscilla).

These references to ‘us’ and ‘them’ recur throughout our interviews. According to social identity theory (Tajfel and Turner, 1986, p. 7; Gloria et al., 2010; Edara, 2020), social identity defines a person’s sense of who they are, and their belonging, based on their group membership that gives individuals a source of pride and self-esteem, be it social class, group, or community. When people are categorised into groups, ‘us’ and ‘them’, such categorisation leads to stereotyping and prejudiced attitudes that can result in racism and hostility. Self-categorised by their nationality, as international Asian or Chinese students, who revered for their economic contributions yet are also ‘temporary’ migrants and thus outsiders thereby create the nuanced multiplicity of ‘us’ and ‘them’ social identity issues. When international students who are temporary migrants are stigmatised as ‘outsiders’ by mainstream media (see ABC Four Corners, 2019), this then publicly creates negative biases against them. For June and Shamim, the language of PM Morrison was ‘detrimental’, and the messaging further reinforced the impression that international students were indeed ‘outsiders’ (June) that added to conscious or sub-conscious bias and discrimination.

First, Australia’s deteriorating relations with China also had an impact on international students from mainland China and other ‘visibly’ – based on physical appearance – Chinese Asian Australians. The public had increasing feelings of suspicion and mistrust reinforced against the Chinese, including international students (Medcalf, 2019; Kassam and Hsu, 2021). During the COVID-19 outbreak, there has been a surge in racism, abuse and hate crimes against Asians in Australia (Chiu, 2020; Razik, 2020; Zhou, 2020). Furthermore, Australia’s alliance with the US and support for Hong Kong and Taiwan also created frictions amongst Chinese students (Klintworth, 2000). Elvina said: ‘amidst the political sensitivity and animosity’, non-mainland Chinese international students in Australia ‘were finding it difficult to uphold their own individual identity’. For Elvina, expressing their views in public amidst mainland Chinese international students were making their own sense of ‘belonging’ to Taiwan challenging. Similarly, Su had to ‘live in a more censored world, pretend[ing] to be…pro-China when with Chinese students’. Or Elvina, who says she ‘cannot be myself in front of them because … I have to say what they want me to say, because they are all pro-Chinese government’. These co-ethnic tensions are not normally captured by the mainstream media that depict all Chinese, or even all Asians, as one single cohort, when students from Hong Kong or Taiwan, for example, can be ‘outsiders’ even within their pan-Chinese cohort (Yee, 2021; Chen, 2023).

Second, international students, especially ‘those looking Chinese’ (interviewees’ words), have experienced incidents of racism and discrimination during the spread of the virus (see also Biddle et al., 2020; Clark et al., 2020; Devakumar et al., 2020; Krishnan, 2020; Kassam and Hsu, 2021; Sojo and Bapuji, 2021). Our data include two international students who were victims of unprovoked racially motivated physical attacks in Melbourne in April 2020 (Giles, 2020; Sakkal, 2020). One of the victims said she never felt welcomed in Australia. Although not self-identified as ethnic Chinese, both the perpetrator and the Australian media—reported in The Age and Canberra Times—depicted her as Chinese, based upon her appearance. Whilst appreciating support and accolades from the mayor and senior managers of their university later, the two students felt ‘there [were] a lot of words, not enough actions’ (Pam). No by-standers offered any assistance, nor did anyone ask if they were alright or needed help, or wanted to call an ambulance. At a more practical level, the international students feared calling an ambulance because of the cost, as, ‘the first thing they asked was for money…$200 on the spot’ for the service (Pam). Yet, despite experiencing a racially motivated physical attack in their first year at university, they carried on with their studies and continued living in Australia. In fact, in the second year, one gained an internship and the other a job. Pam has also stepped up at the university to focus on problems that international students faced during the pandemic, in an ‘affective leadership’ role, that is ‘connect with and influence other people’.

Third, those interviewees who did not face direct racial attacks nonetheless reported feelings of fear and anxiety in public. Even prior to COVID-19, such racist experiences were not new. Su recalls incidents of people ‘yelling, spitting, someone even smashed a glass bottle’ at her. For Su, the ‘anti-China Wuhan virus’ brought ‘back the core issues’ of the ‘outsider’ status in Australia (see Zhou, 2020). Further, when the PM’s ‘go back home’ statement was released in March 2020 (Gibson and Moran, 2020), it was difficult for international students who had just arrived to ‘just go home’. Outbound flights were not readily available and were overly expensive. Shamim (Pakistan, 48) said: ‘it was stressful … confusing, how to return home’ when the airports back home were also closed due to health concerns. Equally, there was a great deal of confusion for inbound students. Encouraged by universities, international students strategized their travels via various third country routes (Choudhury, 2020; Haugen and Lehmann, 2020; Ross, 2020). Su, for example, flew via Tokyo and Hong Kong before 1 February 2020, before catching the last flight into Melbourne. Similarly, Elise flew from China via Thailand in March 2021. Yet, when they landed in Australia, their peers and teaching staff were more concerned about the public health concerns and the spread of the virus upon their arrival than their efforts to return in time.

The foregoing narrative emphasises the stressful temporal–spatial juncture, where mobility is simultaneously transient, temporal and transitional amidst uncertainty and restrictions. Temporal means here that time is fluid, and they were caught in neither being here nor there, not belonging and yet present and studying in a web of chronomobility. Punctuated by the media depiction of the ‘Wuhan’ virus and the PM’s perceived unwelcoming message that intensified the ‘outsider’ status also caused disruptions of their planned arrival, prompt commencement of studies and emotional anxiety, all of which affected other spheres of their lives.

### 3.2 Economic vulnerabilities

For most international students interviewed economic vulnerability was the core issue: how to support themselves during the pandemic. Of the 15 respondents, three had sufficient savings to rely upon from their scholarships, and two had parents who continued to support them. International students commonly find jobs as casuals to pay rent and living expenses during their studies in Australia. Amar, Shamim, and Pam all from different countries thought the lockdowns in Melbourne would be over soon and did not justify a return. June said: ‘at first, I did not think that this pandemic would last this long. If I knew, … I would have just packed, … going back to Korea’. For Elise the justification was that ‘COVID-19 is everywhere … even in China … [in Australia] sometimes there’s a small outbreak, so I think there’s no point to go back. And the most important thing is, I’m doing the study in Australia. I want to optimise my experience, so I chose to stay’. Hence staying behind was the best choice for her and others too, as they were focussing on completing their degrees, making applications for further studies, and extending their student visas. Others thought that they could find jobs, since borders were closed, and Australian businesses would look for onshore candidates (Abir and Priscilla).

Casual workers are allowed to work up to 20 h a week, with the hospitality sector being the largest employer of international students as casual workers (International Education Association of Australia, 2021). Yet the hospitality sector was hit hard by the pandemic: students were the first to lose their jobs owing to their ‘casual’ status. Ineligible for Australian subsidies such as the *Job-Keeper* or the *Job-Seeker* payments in 2020 (Australian Government, 2020), international students were disproportionately affected by the downturn. This temporal shift from ‘cash cow’ to residual temporary workforce further perturbed international students’ feelings of their status and objectification in Australia. According to Elvina, “when things got tough, the ‘cash cows’ were rapidly branded as ‘temporary’ migrants,” thus ‘absolving the government and universities from concern for their well-being’. Pam says those jobs were important particularly during the pandemic because ‘we [students] cannot depend on the university … because they would not waive the fees and we are paying full fees to watch teachers on Zoom’. Kevin elaborated that, although ‘international students pay higher fees…, [we are] the last ones being accorded services … unfortunate and pathetic’.

Many middle-class international students rapidly became the urban poor during the pandemic, queuing up for free food at the CBD foodbank or accepting hardship allowances from universities and the community. Pam expressed this predicament: ‘we were literally just given the food bank … everything else was … you just do what you can’. The perceived impression was ‘you know, we are only worth our money’. This was so much so that going to the foodbank became a socialising event in their lives, since ‘almost everyone I know or am friends with has been through the food bank once’. Yet knowing that many international students come from middle-class families back home, queuing for food handouts, relying on or asking for support from universities was emotionally challenging for these temporary migrants who were promised upward social mobility and privilege through education but were now struggling. Shamim commented: ‘where they [students] come from and where they are placed during COVID-19’, was disheartening; the food queues were ‘both shocking and disturbing as … this was not something in our minds when you come to Australia’.

Many were relying on families overseas for financial aid to survive and were coping with new life processes that shifted from self-sufficiency to dependency, and feelings of angst on their plight. Ibrahim, Priya and Kevin felt guilty having to ask their parents who, like most other people, were also facing hardship at home during the global pandemic-economic downturn. Voicing this anxiety, Ibrahim said it was ‘disconcerting, because, as students, we are hoping to be independent and be self-supportive, making contributions to funds already expended on their [i.e., our] education’. For Su, the university one-time grant saved her life, because ‘… I was so broke … I was living on a single digit last year (late 2020)’. Abir revealed that the university grant was a blessing, as he and his wife had both lost their jobs and had no other income source.

Yet some Australian employers in fact added to these economic woes experienced by international students, using against them their temporary casual work status. They were thus the first to lose their jobs when the pandemic hit the vibrant hospitality followed by other business sectors relying on casual labourers. However, their jobs were also the ones to be filled during the labour shortage by Australian nationals, making international students suddenly quite dispensable. Emphasising this, Huu explained that some employers, for instance, were advertising weekly working hours as 21–22 h in vacancy notices, which automatically barred international students, who were legally allowed only 20 h. Similarly, law firms only recruited people who spoke Asian languages, and were citizens or permanent residents, overlooking international students with similar skills (Huu), which again refocussed their temporality – they are not belonging, yet also being present in Australia.

### 3.3 Transition to online learning

With the onset of COVID-19 health restrictions, universities rapidly switched to digital on-line education (Watermeyer et al., 2021). For many students, studying from home and online was highly challenging. June and Priya explained how the confined spaces where they lived further blurred the boundary between study and life balance, a liminal suspended time-space. June, working from home in a studio, alone, reflected on her experience: ‘if you turn around, you have your bed, and if you turn that side, you have your PhD work. So, I feel like I’m working all day without really focusing on my work’. In this context of ‘temporality and space’ experience, she lost her tempo of life, which then affected her motivation to study. For Priya and Gilbert (Philippines, 28), who shared accommodation with other family members in their own countries, ‘getting on top of each other’, ‘having some [familial] conflicts’, and ‘fulfilling family obligations’ were altogether taxing for them emotionally. The shift to financial dependency and to lack of autonomy, life in small, confined spaces, and differences in the ongoing yet also suspended tempo of life processes jointly slowed their motivation and progress in their studies.

## 4 Coping mechanisms and strategies

Trapped in a temporal–spatial zone which COVID-19 created, all participants felt vulnerable owing to the precarity described in the earlier sections: as ‘outsiders’, impecunious and space-confined amidst uncertainty. The COVID-19 Pandemic triggered an increase of racist and xenophobic incidents against people of (perceived) Chinese or Asian origin. Interviewees said amidst the temporality, uncertainty and precarity, COVID-19 related racism and uncertainty led to stress worry. In transactional stress and coping theory (TTSC), Lazarus and Folkman (1984) analyse the effect of stress and coping strategies on mental well-being. *Stress* is considered a transaction between an individual and the environment, whilst *coping* as the relationship between overcoming vulnerability towards better mental health for individuals (Folkman et al., 1986; Lee and Roberts, 2018). Despite these various challenges, international students developed their own coping strategies and mechanisms.

### 4.1 Finding out what works—meditation and virtual escapism

The two broad categories of coping strategies are problem-focussed seeking external support, and an emotion-focused internal state to reduce stress (Lazarus and Folkman, 1984). Addressing anxiety and helplessness, loneliness and COVID-related racism, international students, urban middle-income-migrants, resorted to devising various means to alleviate their stress related problems. June took to meditation when she felt she had to deal with events beyond her control. Meditative state or meditation is a form of withdrawal of the senses to handle stress, by switching off from the outside world and turning the gaze inward, something akin to a trance. She meditated morning and evening whilst listening to podcasts to relax. She observed that ‘sometimes your brain is too active, and you cannot even focus on meditation’ yet ‘meditation really helps alleviate anxiety’. Likewise, Ibrahim adapted by ‘accepting reality that nothing is in my hands, just loving what I am doing, [since] there is no other option, that is how I am coping’. Recognising the need to remain positive, Huu began ‘disengaging from social media news’ recognising it as a ‘stressor’. For solace, Shamim developed a new habit: ‘getting up early in the morning to pray before starting off my day’. This morning routine helped her reduce feelings of helplessness and accept the reality was out of her control. Focussing instead on gratitude, Shamim found meaningful positivity in tough times.

Other participants find their place of contentment in an alternative reality to cope with the disruption and stress caused by immobility and isolation during the pandemic. In other words, by being trapped in a temporal and spatial zone, they find their ways to resist being affected by the negative consequences of the pandemic. We call such a coping strategy as ‘virtual escapism’, and it is a reactive act in which participants try to disconnect from what is happening in their immediate daily lives by immersing themselves in an alternative reality that does not physically exist.

Reading *manga* can be a way into an alternative reality. For Su *manga* (Japanese graphic novels) or music helped her to release stress. Elvina and Huu also found *manga* a form of virtual escapism, breaking away from the pandemic reality in Melbourne. Amongst them, Elvina is the most passionate about reading *manga* and she also includes watching *anime* and playing games in many forms (online, offline, and mobile) as strategies to ‘cope with my [her] stress and depression’ and ‘to relax my [her] brain’ because these things do not require her ‘brain power’ and she can just ‘take a rest’. For Kevin, playing online games is not necessarily his way to relax his brain, but to connect with his brother who lives with his family in Indonesia. Watching movies, dramas and documentaries is a form of virtual escapism. Huu binge-watched Netflix Korean dramas and films such as *Squid Game* and *Parasite.* Immersing themselves in different distracted spaces to self-reflect, in order to process their temporal continuity as transients, gave these international students some meaning and stability in their suspended lives.

### 4.2 Making routine habitual for personal development

Lazarus and Folkman (1984, p. 141) define *coping* as ‘constantly changing cognitive and behavioural efforts to manage specific external and internal demands that are appraised as taxing or exceeding the resources of the person’. Therefore, to cope, individuals devise ‘coping mechanisms’ (appraising the crisis causing stress) and ‘coping strategies’ (finding the ability to handle the situation) through adaptation (Stephenson and DeLongis, 2020). Whilst meditation and virtual escapism were de-stressors and coping mechanisms, routine habitual targets, self-improvement, networking and seeking help became part of coping strategies.

Participants used regular exercise as a common coping strategy. Priscilla, who had never run before, started training to run a half-marathon, enjoying the endorphin energy burst. Like Priscilla, Amar started taking part in cycling events with her friends or going for long walks. Elise devised a ‘good morning’ routine: ‘wake up early at 7 a.m., do a 10-min meditation, some reading for 30 min, exercise for 30 min, [followed by] a good breakfast’, all to improve her productivity.

Others turned around their pandemic misfortune and isolated time for personal development and added skill sets. For instance, Elvina took up an online Japanese language class with a private tutor in Osaka. Ibrahim took up a new computer programming language to expand his knowledge for future potential jobs. Finding comfort in writing so as not to give way to sadness, Su finished writing three pieces of fiction during her thesis: ‘Looping Souls, Sad Souls is a random way of coping … that kind of helped’ her to keep on going, and to ‘… kind of recalibrate into the new normal’. Similarly, Amar, a psychology student, started ‘Journaling a lot … Like I have this thought … channel where I jot things … it’s a sort of catharsis, you feel a lot better’. Rather than requesting counselling services and waiting five days, journaling helped Amar in ‘clearing headspace, creating pathways for mental well-being’. Through these many ways, the participants were trying to detach themselves to find their own tempo of life and space, a duality of being here and there, fluid but also stationary.

### 4.3 Maintaining relational connectivity with families and close friends

One of the worst hardships was familial distance, or ‘the tyranny of distance’, during the pandemic. Many had not seen their families for 2 years, given the extended 2021 lockdowns. They said that the first thing they would like to do when the borders re-open was to see their families. Thus, prioritising families’ presence somehow was a regular feature in their day-to-day suspended lives. Abir remarked:

*Every day I think that now, if something happens to my father, I will pack a bag and go back forever … I will not be coming back, because money can’t buy happiness… And it’s very important … our happiness, our mental happiness is more than the financial happiness … harsh.*

### 4.4 Networking—virtual social connections

Yet even whilst physically isolated, students were actively engaging with their peers online. Social media was by far the most common tool to remain connected with families and friends. Indulging in humour as a ‘feel-good’ factor, some participants took to ‘sharing memes, posting jokes and fun things’ (Su; Huu)—humorous images were a way to remain cheerful. Also, memes and jokes were ‘just trying to keep people through the 14-day quarantine that they have to go through and … to keep people’s hopes alive … of coming back into Australia, … continuing to study’ (Su). Humour and laughter are often recognised as a re-structuring of a situation by making it less threatening and a concomitant release of emotion (Kuiper et al., 1995; Abel, 1998).

For many, the second lockdown from 9 July 2020, which lasted for 112 days in Melbourne, was the most challenging. Stringent health regulations limited opportunities for social contact. To help reduce their day-to-day stress, Elise and her friends would ‘connect over a coffee catch-up’ with others living within the 5-km allowed space for exercise. Priscilla, who was ‘never into coffee before COVID’, now became addicted to coffee and coffee catchups. For Priscilla networks and friendship during the pandemic were high priority:

*the most important thing was networks … a strong international student network here … even though you sometimes don’t feel welcome in a foreign country, but If you have that very solid and strong network … that is so powerful.*

These social networks and community activities motivated the participants to stay positive and active. Kevin and Gilbert began organising online events, connecting newcomers, and taking up volunteer leadership roles such as youth ambassadorships or fund-raising for isolated communities, building their confidence and a sense of belonging.

Pam’s story is inspirational for other international students. After the racist attack in March 2020, Pam learned to turn negative energy into positivity by actively engaging in student activities. She decided to use her ‘emotions productively … rather than seeking pity’. Kevin found ‘more people contributing to their own growth … but also a way to make friends, feel you are a part of something … University has not done much to support us, that’s why we have chosen this forum’. Kevin felt that students’ voices were not being listened to during the university-led ‘consultative’ forum. Most interviewees found student-led forums more helpful in overcoming loneliness and an increased sense of belonging than university endeavours.

### 4.5 Counselling services

Most Australian universities offered counselling services for all students, recognising that COVID-19 had created significant disruptions, challenges, and vulnerabilities. However, for many Asian international students, for cultural reasons, talking about mental health issues was challenging. Su explained that Asians generally “do not use the terms ‘mental health’, ‘depression’, and anxiety and things like that.” She added that mental health issues are ‘something [perceived] as a sign of weakness… [you are] not supposed to cry, [be] missing home’. Initially, international students abroad were not eligible for counselling services. Priya expressed her strong disappointment: it ‘sucks, because, you know, they [overseas] are the people need help the most’ and a ‘shame that Australia cannot take care of international students, the largest cohort overseas … the strongest contingent’. To seek mental health support, international students abroad turned to the student union to request online Counselling and Psychological Services (CAPS). Yet the only available sessions were early morning. Priya elaborated: ‘[would] any one in their right mind to get up at 5.15 a.m. in the morning for a counselling session … to talk about the most morbid thing happening in their life that can save you…?’

On the one hand, this shows universities’ reaching out, but, on the other, their inability to appreciate the different time zones and how these affected international students both learning online and in their mental health, which heightened lack of attention to detail. This was made evident by international students who discussed some having to switch to online learning overseas during the lockdown. Melbourne’s official working hours did not consider Asian time zones: being either very early or late-night times, both considered inappropriate. Yet more international students who also used the services during the pandemic in Melbourne, questioned the accessibility, quality, and adequacy of the counselling services.

### 4.6 Counselling services—inadequate counselling strategy

They believed that the services were ‘unprepared’ (Pam) for serious racism cases and ‘less than expected’ (Elise), since often services seemed to be provided by inexperienced counsellors or sometimes by graduate trainees. Some reported that university counselling services were meant to ‘solve the problem’ (June and Pam), but often students were referred to various other departments when requiring special consideration, such as applications for late assignments. Students also felt that university free counselling services were not tailored to meet the unique needs of students in different life stages. For example, June felt that counsellors ‘do not really understand the constraints of PhD [students] because they do not have lived experiences … first-hand experience of doing a PhD … They just do not understand the life of HDR [higher degree research] students’. Lack of understanding led many international students—including June—opting not to continue with their remaining counselling sessions.

A key concern was that language was identified as a barrier in counselling services. All international students are required to sit English language proficiency and fluency tests (ELICOS) prior to being accepted for higher education in Australia. All interviewees are fluent English speakers but found it difficult to translate their emotions into medical terminology in English or find equivalent English words expressing their emotions and on-going vulnerability. One ‘cannot quite communicate what one can [only] say in ethnic languages … particularly when one is depressed’ (Elise). According to Elise, ‘CAP counselling services are not doing justice, because 80% of international students are ethnic students, and 80 per cent of counsellors are Australian (not Asian)’. Su considered that it was the counsellors’ assumed superiority that contributed to international students’ low return rate to counselling sessions. However, in other international students’ minds, it was the language and cultural or ethnic insensitivity that they believed was a cause of inadequacy in quality counselling services. The demographics of counsellors and students are unknown because this data is not available. More research needs to be done on the causes of distrust in the quality of university counselling services.

## 5 Conclusion

Our data revealed that, despite the various socioeconomic vulnerabilities they faced during COVID-19 in Australia, many international students actively adopted to strategies to sustain their physical and mental wellness during the global pandemic. This was particularly so in metropolitan Melbourne, which had the longest and multiple lockdowns. International students, being considered ‘outsiders’ and, at the same time, a ‘disposable’ workforce, adopted various coping mechanisms, including spiritual meditation, physical exercise, new skills acquisition, virtual escapism, or active community participation. Social networks and connections with their peers and families were identified as one of the most important aspects for overcoming mental stress.

Instead of viewing international students through the ‘education commodity’ lens, a major source of revenue for universities or viewing them as victims of exploitation, this study finds Robertson’s middling class and chronomobility as a more useful understanding of their experiences. It shifts away from demand and supply and gravity model theorists and researchers (Open Doors, 2005; HESS 2008; Abbott and Silles, 2016; Firang, 2020). The past decade—in broad migration studies cutting across single and inter-disciplinary studies of ethnic and racial studies, including the pandemic period—has seen the emergence of a ‘temporal turn’ in the 21st century causing scholars to rethink migration experiences and address new interrelated questions on temporalities and mobilities and the affective experiences.

Robertson’s chronomobility framework interrogates the underlying links between emotional experiences of time, day to day, in migrants’ work, leisure and social experiences. International students in their early stage of middling class and at their specific life courses progressions, faced one of the longest lockdowns in Melbourne metropolitan area. Whilst individuals’ capacities differed, their temporal and spatial environments were the same, given the pandemic conditions in Melbourne. A combination of these different endogenous and exogenous factors would make their individual experiences unique. COVID-19 restrictions blurred their conceptions of time and space when their lives were at once suspended and yet also on-going in time. The study-life boundaries were reconfigured and recalibrated, as if they were constantly on the move, simultaneously temporal and transient. Under these pandemic coping mechanisms, students adjusted to new bodily regimes in odd times and confined and liminal space, so as to stay focussed on their studies during this period of uncertainty. Not all succeeded in recognising their stresses within their own coping mechanisms and coping strategies. In fact, a few continued to struggle. However, most of the students interviewed for this study presented an elevated level of resilience and positivity, including those who were victims of physical racial attacks in central Melbourne. Faced with comparable challenges, many found linear multiplatform jobs and supported one another.

The existing hostile attitudes towards Asian/Chinese international students, coupled with inadequate and insensitive institutional support, seemed to deepen their fear and anxiety about studying and living in Australia. Although student-led support communities were positively evaluated, there were serious concerns about university-provided counselling services. Shortcomings identified ranged from services not being tailored to student needs, inadequate staff training and inexperience in handling COVID-19 racism, non-Asian counsellors being used, and inadequate cultural sensitivity training which led to perceptions of assumed superiority amongst counsellors towards students.

Our findings tell us that students’ lived experiences of mobile temporality during the pandemic and their coping mechanisms in the socio-economic education-migration nexus. Students’ ‘temporary’ status and their transient transnational fluidity during the pandemic, together with their coping mechanisms demonstrate their resilience and resourcefulness. These complex multi-directional transitions and temporalities provide the education migration sector with an opportunity to enhance services for international students. Migration—student visas, temporary work visas—are often the entry point for multistep migration pathways. Focussing merely on demand and supply or gravity model theorists that view international students through the ‘commodity’ lens fails to appreciate their lived experiences particularly during the COVID-19 pandemic: experiences that combine geo-politics, the shifting and fluid links between temporalities and mobilities envisaged during closed international borders. Today’s international students are potentially Australia’s future skilled migrant workforce and/or active citizenry. Their pandemic experiences and narratives will inform and influence future international students and skilled migrant cohorts within their transnational social networks. Rather than viewing them as ‘commodities’ or ‘cash cows’ or ‘victims’, Australian policymakers, educators and academics need to address issues of institutional support that is sensitive to the needs of international students, and which empowers them to build stronger student communities and tap the talent and knowledge resource they bring to Australia.

## Data availability statement

The original contributions presented in the study are included in the article/supplementary material, further inquiries can be directed to the corresponding author.

## Ethics statement

The studies involving humans were approved by University of Melbourne Human Ethics Committee. The studies were conducted in accordance with the local legislation and institutional requirements. The participants provided their written informed consent to participate in this study. Written informed consent was obtained from the individual(s) for the publication of any potentially identifiable images or data included in this article.

## Author contributions

JS provided the conceptual framework and the design of research methods, and conducted five interviews. SD and JO conducted additional five interviews each. All authors contributed to the article and approved the submitted version.
